# Critical points and effectiveness of prophylactic C4/5 foraminotomy to prevent C5 palsy after posterior cervical spine surgery

**DOI:** 10.5152/j.aott.2021.21239

**Published:** 2021-11-01

**Authors:** Kazunari Takeuchi, Toru Yokoyama, Kanichiro Wada, Gentaro Kumagai, Eiji Sasaki, Yasuyuki Ishibashi

**Affiliations:** 1 Department of Orthopedic Surgery, Hirosaki University School of Medicine, 5 Zaifu-cho, Hirosaki, Aomori, Japan; 2 Department of Orthopedic Surgery, Odate Municipal General Hospital, 3-1 Yutaka-cho, Odate, Akita, Japan

**Keywords:** Cervical myelopathy, C5 palsy, C4/5 foraminotomy, Laminoplasty, Posterior fusion, Cross-sectional area

## Abstract

**Objective:**

The aim of this study was to clarify the cut-off values of the spinal canal parameters as risk factors for C5 palsy after posterior cervical spine surgery with and without foraminotomy.

**Methods:**

One hundred three consecutive patients (67 males, 36 females; mean age = 66 years, age range = 27–87 years) with cervical myelopathy who underwent posterior cervical spine surgery at our institution were retrospectively reviewed and included in the study. The first consecutive 69 patients who underwent posterior cervical spine surgery with prophylactic bilateral C4/5 foraminotomy were designated as the F (+) group. The subsequent 34 consecutive patients who underwent posterior cervical spine surgery without prophylactic bilateral C4/5 foraminotomy were designated as the F (–) group. All patients were then divided into four subgroups. In the F (+) group, patients with C5 palsy were designated as the F (+) P (+) subgroup (n = 13), while those without C5 palsy were designated as the F (+) P (−) subgroup (n = 56). In the F (−) group, patients with C5 palsy were designated as the F (−) P (+) subgroup (n = 5), while those without C5 palsy were designated as the F (−) P (−) subgroup (n = 29). Receiver operating characteristic curves were used to investigate the cut-off values of the spinal canal parameters for the development of postoperative C5 palsy. The assessed spinal parameters were the gutter positions (GP), laminar inclination angles (LIA), and postoperative cross-sectional areas (CSA) of the dural sac. The risk ratios (RR) of the spinal canal parameters as risk factors for C5 palsy were evaluated.

**Results:**

The incidence of C5 palsy was similar between the F (+) group (18.8%) and the F (−) group (14.7%). The cut-off values for each spinal canal parameter in the F (+) group (GP: 0.82–0.84, LIA: 58.9–62.4°, and CSA: 189.5–200 mm^2^ ) were similar to those in the F (–) group (0.81–0.89, 61.7–62.5°, and 197.5–199.5 mm^2^, respectively). In the RR results for C5 palsy, the LIA was highest in both groups. The F (+) P (−) subgroup had significantly larger mean CSA at C4/5 and C5/6 (202.3 mm^2^ and 200.9 mm^2^, respectively) than the F (−) P (−) subgroup (177.3 mm^2^ and 178.9 mm^2^, respectively) (P = 0.0181 and P = 0.0277, respectively). Prophylactic C4/5 foraminotomy did not specifically prevent postoperative C5 palsy due to foraminal stenosis at C4/5.

**Conclusion:**

C4/5 foraminotomy should not be recommended for avoidance of C5 palsy. Although the bony spinal parameters were similar between the F (+) and F (−) groups, the CSA in the F (+) group was significantly than that in the F (−) group in the patients without C5 palsy.

## Introduction

There are many reported risk factors for C5 palsy after posterior cervical spine surgery. One frequently reported risk factor for postoperative C5 palsy is posterior shift of the spinal cord.^1-^^6^ Therefore, the cross-sectional areas of the spinal cord and the dural sac in the cervical spine might be larger in patients with postoperative C5 palsy than in patients without postoperative C5 palsy. Although some studies have reported that prophylactic C4/5 foraminotomy prevents C5 palsy after cervical laminoplasty (LP),^7-^^9^ others have reported that C4/5 foraminotomy does not prevent the development of C5 palsy after LP.^10–12^ Therefore, it remains controversial whether C4/5 foraminotomy effectively prevents postoperative C5 palsy. Furthermore, the cut-off values of the bony spinal canal parameters and the postoperative cross-sectional area of the dural sac (CSA) for the occurrence of C5 palsy after posterior cervical spine surgery with and without C4/5 foraminotomy remain unknown. Thus, we do not know the laminoplasty procedures that should be paid attention to for avoidance of postoperative C5 palsy during LP with and without posterior fusion.

The purpose of the present prospective study was to clarify the critical cut-off values for the spinal canal parameters and assess the effectiveness of C4/5 foraminotomy in preventing postoperative C5 palsy. The assessed spinal parameters included the gutter positions (GP), laminar inclination angles (LIA), and CSA.

## Materials and Methods

### Patients

From 2011, 103 consecutive patients (67 males and 36 females) with cervical myelopathy underwent posterior cervical spine surgery at our institution and were included in the present study (follow-up rate: 93%). The mean age at the time of surgery was 66 years (range, 27-87 years). The first consecutive 69 patients underwent posterior cervical spine surgery with prophylactic bilateral C4/5 foraminotomy and were designated as the F (+) group. From 2015, the next 34 consecutive patients underwent posterior cervical spine surgery without prophylactic bilateral C4/5 foraminotomy and were designated as the F (–) group. Table 1 summarizes the characteristics of the two groups. All patients with ossification of the posterior longitudinal ligament (OPLL) who underwent LP had K-line (+) OPLL.^13^ PDF was used for patients with K-line (–) OPLL in the neck-flexed position^14^ or cervical spondylotic myelopathy (CSM) with malalignment. All patients received intraoperative transcranial stimulation motor evoked potential monitoring of the deltoid and biceps brachii muscles.

All patients were divided into four subgroups. In the F (+) group, patients with C5 palsy were designated as the F (+) P (+) subgroup (*n* = 13), while those without C5 palsy were designated as the F (+) P (−) subgroup (*n* = 56). In the F (−) group, patients with C5 palsy were designated as the F (−) P (+) subgroup (*n* = 5), while those without C5 palsy were designated as the F (−) P (−) subgroup (*n* = 29). This study was approved by the institutional ethics committee according to the 1964 Helsinki declaration, and informed consent was obtained from all participants.

### Operative technique and postoperative treatment

**Figure 1.**
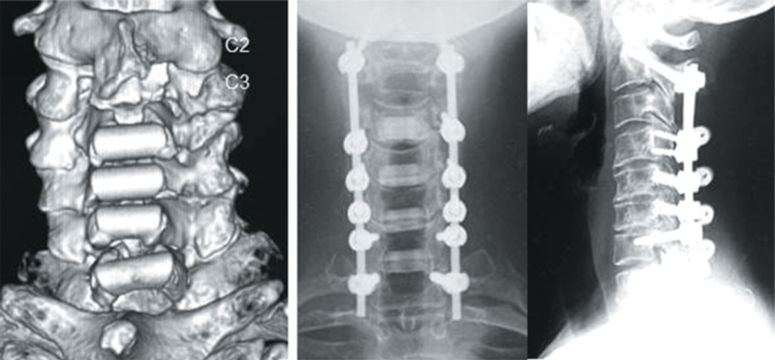
Double-door laminoplasty using hydroxyapatite spinous process spacers at C4-C7 with C3 laminectomy preserving all muscle insertions at C2, and C2-T1 posterior decompression and fusion. **Left**: Anteroposterior three-dimensional computed tomographic image of cervical laminoplasty alone (C3 laminectomy and C4-7 laminoplasty). **Middle**: anteroposterior and **right**: lateral radiographs of the C2-T1 posterior decompression and fusion included C3 laminectomy, C4-7 laminoplasty, and C2-T1 instrumented fusion.

**Figure 2.**
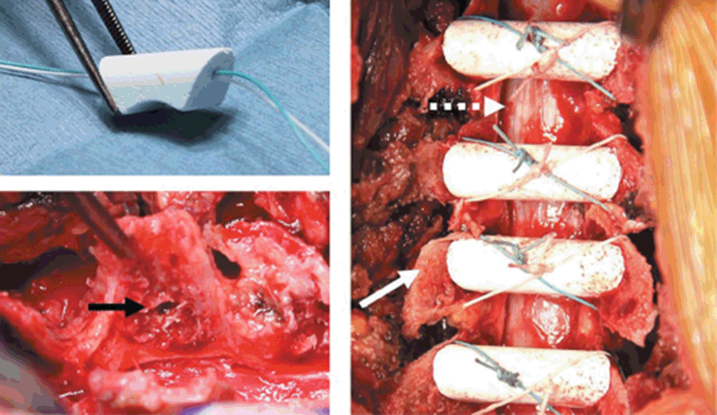
Fixation of the spinous process spacers to the lifted laminae. **Upper**: Hydroxyapatite spinous process spacer with a penetration hole and two non-absorption threads. **Bottom**: A hole (black arrow) made in the lifted laminae using a surgical bar. **Right**: Spacers fixed to the lifted laminae using two non-absorption threads. A white solid arrow: a lifted lamina at C6; white dotted arrow: dura.

**Figure 3.**
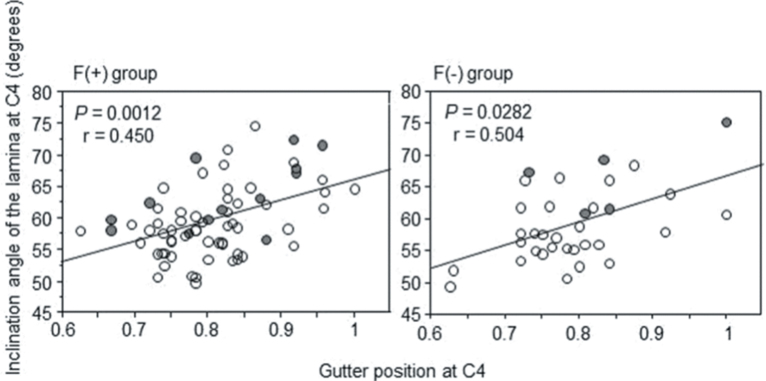
The gutter position was significantly positively correlated with the C4 laminar inclination angle in the Foraminotomy (+) and Foraminotomy (–) groups. Spearman’s rank correlation was used for the statistical analysis. Gray circles: patients with C5 palsy; white circles: patients without C5 palsy.

**Figure 4.**
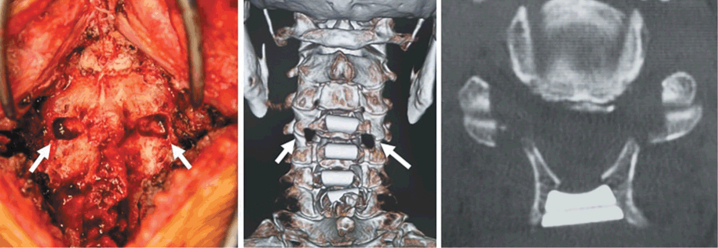
Cervical laminoplasty with prophylactic bilateral C4/5 foraminotomy. **Left**: Photograph showing the foraminotomy (arrows). **Middle**: Anteroposterior three-dimensional computed tomographic image showing the foraminotomy (arrows). **Right**: Axial computed tomography.

LP included C3 laminectomy and C4-7 laminoplasty with the semispinalis cervicis into C2 (Figure 1).^15^ PDF included C3 laminectomy, C4-7 laminoplasty, and posterior instrumented fusion at C2-T1 with preservation of all muscle insertions at C2 (Figure 1).^16^ Spinous process-splitting LP was performed using hydroxyapatite spinous process spacers at C4-C7 (Figure 1).^17^ The spacer had a penetration hole to allow two threads to pass through, and a hole was made in the lifted laminae at C4-C7 using a surgical bar of 2 mm diameter. The spacers were grown on a cross and fixed to the lifted laminae at C4-C7 using two non-absorption threads. The spacers do not come into contact with the neck extension, as the dorsal face of the spacers is round in shape (Figure 2). The GP and the LIA were gradually extended over time in this case series to achieve sufficient postoperative expansion of the spinal cord and the dural sac in both groups (Figure 3). In the F (+) group, bilateral C4/5 foraminotomy was performed immediately before the LP or PDF procedure (Figure 4). Complete decompression of the C5 nerve root was confirmed via the smooth insertion of a micro-spatula into the expanded foramen (Figure 4). None of the patients required postoperative immobilization with a collar. Patients were permitted to sit up or walk within 1 week postoperatively, and exercise was resumed within 1 week postoperatively.

### Assessment of postoperative C5 palsy

Postoperative C5 palsy was defined as new deterioration of muscle strength of the deltoid and/or the biceps brachii as detected by manual muscle testing.^1^

### Relationships between the occurrence of postoperative C5 palsy and the perioperative related factors

Relationships between the occurrence of postoperative C5 palsy and perioperative-related factors, including diseases (CSM or OPLL), age at the time of surgery, and surgical methods (C3-C7 LP or C2-T1 PDF). All patients were divided into two groups based on the age of 66 years (mean age of the population): younger (age less than 66 years) and elderly (> 67 years) groups.

### Spinal canal parameters and preoperative foraminal diameter at C4/5

**Figure 5.**
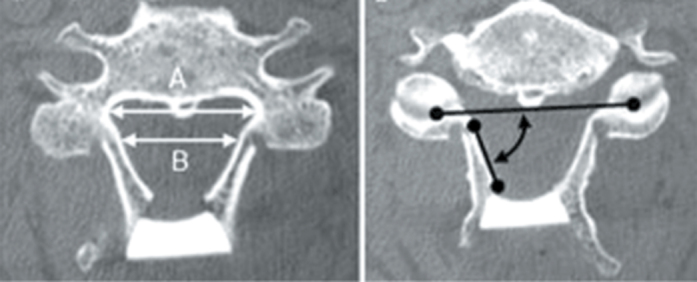
Bony spinal parameters measured on axial computed tomographic images. **Left**: Gutter position. (**A**) Transverse diameter of the spinal canal (**B**) Distance between bilateral gutters. **Right**: lamina inclination angle.

**Figure 6.**
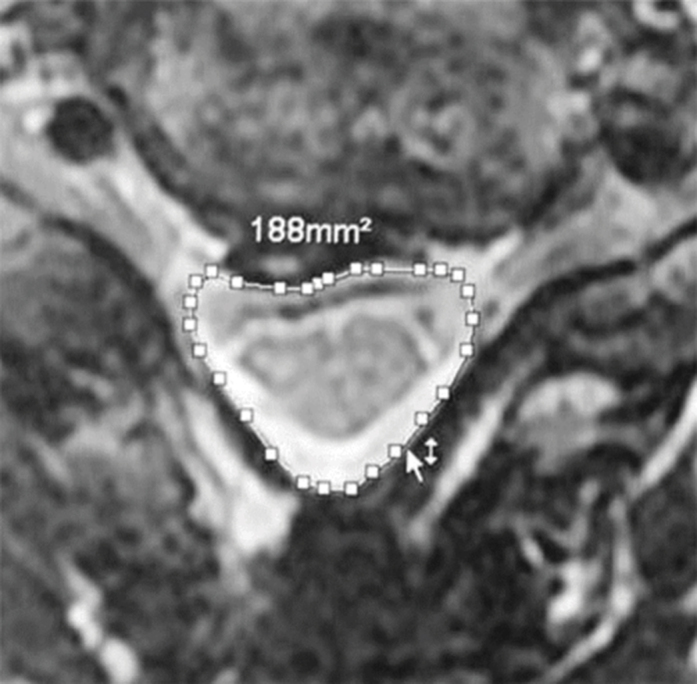
Axial T2-weighted magnetic resonance image showing the measurement of the postoperative cross-sectional areas of the dural sac.

The bony spinal canal parameters were measured on postoperative CT using XTREX VIEW (J-MAC System, Sapporo, Japan).^18^ The GP was defined as the inside distance between bilateral gutters/the transverse diameter of the spinal canal (Figure 5).^18^ The LIA was measured as the angle created by the line between the bilateral facet joints and the line between the rising point of the inside of the lamina and the inside corner of the lamina on the spacer side (Figure 5).^18^ Preoperative transverse diameters of the bilateral foramina at C4/5 were also measured on the axial view on CT.^19–21^ The postoperative CSA at C4/5 and C5/6 were measured on T2-weighted axial magnetic resonance imaging using XTREX VIEW (Figure 6).^18^ The GP and LIA were measured at 2 weeks postoperatively. The postoperative CSA were measured at 1 year postoperatively.

### Neurological outcomes

The pre- and postoperative Japanese Orthopaedic Association (JOA) scores and the recovery rate (%) of the JOA score were investigated in all patients. The recovery rate of the JOA score was calculated as: recovery rate (%) = (postoperative JOA score **–** preoperative JOA score)/(17 **–** preoperative JOA score) × 100. The follow-up period for these neurological outcomes was 1 year in all cases.

### Statistical analysis

The cut-off values for the occurrence of postoperative C5 palsy were assessed using receiver operating characteristic (ROC) curves for the following three factors in the F (+) and F (–) groups: GP, LIA, and CSA. The risk ratios (RRs) of the risk factors that were set by each cut-off value for the occurrence of postoperative C5 palsy were statistically analyzed in each group. Fisher’s exact test, Mann–Whitney *U*-test, and Spearman’s rank correlation were used for the statistical analysis. Differences with a *P* value of less than 0.05 were considered statistically significant.

## Results

### Incidence of postoperative C5 palsy and neurological outcomes

Of the 69 patients in the F (+) group, postoperative C5 palsy occurred in 13 patients, giving an incidence of 18.8%. Of the 34 patients in the F (−) group, postoperative C5 palsy occurred in five patients, giving an incidence of 14.7%. The incidence of postoperative C5 palsy was similar in the F (+) and F (−) groups.

The mean preoperative JOA scores, postoperative JOA scores, and recovery rates of the JOA score were similar in the F (+) (10.2%, 13.1%, and 43.7%, respectively) and F (−) groups (10.0%, 13.2%, and 47.7%, respectively).

### Relationships between the occurrence of postoperative C5 palsy and the perioperative-related factors

The disease, age at the time of surgery, and surgical methods were not related to the occurrence of C5 palsy (Table 2). The mean ages of the patients were similar between with (66.1 years) and without C5 palsy (65.4 years).

### Cut-off values of the spinal canal parameters for the occurrence of postoperative C5 palsy

**Figure 7.**
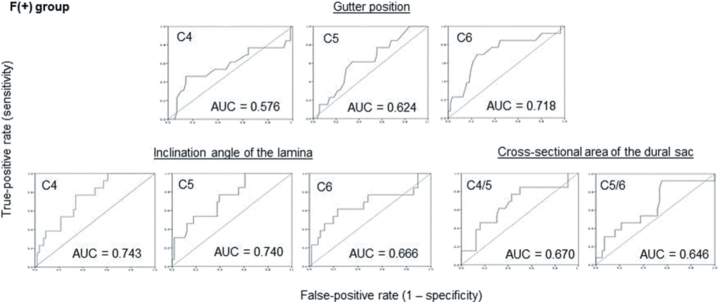
Receiver operating characteristic curves in the F (+) group. F (+) group, patients who underwent spinal surgery with foraminotomy; AUC, area under the curve.

**Figure 8.**
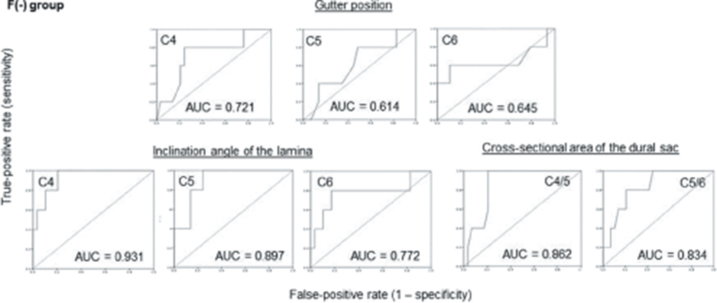
Receiver operating characteristic curves in the F (–) group. F (–) group, patients who underwent spinal surgery without foraminotomy; AUC, area under the curve.

Table 3 summarizes the cut-off values and areas under the ROC curves (AUC) for the spinal canal parameters for the occurrence of postoperative C5 palsy in the F (+) and F (−) groups. In the F (+) group, the AUC results showed that several cut-off values for the spinal canal parameters had moderate accuracy (0.70-0.90) for the prediction of postoperative C5 palsy.^22^ In the F (−) group, the AUC results showed that many cut-off values for the spinal canal parameters had moderate accuracy (0.70-0.90) or high accuracy (0.90-1.0) for the prediction of postoperative C5 palsy.^22^ The ROC curves of these parameters in each group are shown in Figures 7 and 8. The cut-off values for each spinal canal parameter in the F (+) group were similar to those in the F (–) group (Table 3).

The RR for C5 palsy occurrence in each group is shown in Table 4. The RRs of the LIA at C4-C6 in the F (+) group, LIA at C4 and C5, and GP at C6 in the F (−) group were higher (Table 4).

### Relationships between C4/5 foraminotomy and the spinal canal parameters

In the 18 patients with postoperative C5 palsy, the spinal canal parameters (GP at C4-C6, LIA at C4-C6, and CSA at C4/5 and C5/6) in the F (+) P (+) subgroup were similar to those in the F (−) P (+) subgroup (Table 5). In the 85 patients without postoperative C5 palsy, although the bony spinal canal parameters in the F (+) P (−) subgroup were similar to those in the F (−) P (−) subgroup, the F (+) P (−) subgroup had significantly larger mean CSA at C4/5 and C5/6 (202.3 mm^2^ and 2009 mm^2^, respectively) than the F (−) P (−) subgroup (177.3 mm^2^ and 1789 mm^2^, respectively) (*P* = 0.0181 and *P* = 0.0277, respectively) (Table 5). Therefore, although bilateral C4/5 foraminotomy did not contribute to the enlargement of the bony spinal canal parameters, foraminotomy effectively increased the postoperative CSA in the patients without postoperative C5 palsy.

### Relationships between the preoperative transverse diameter of the foramen at C4/5 and prophylactic C4/5 foraminotomy

Prophylactic C4/5 foraminotomy did not specifically prevent postoperative C5 palsy due to foraminal stenosis at C4/5, as the preoperative transverse diameter of the foramen at C4/5 in the F (+) group was similar to the diameter in the F (–) group in patients with and without C5 palsy (Table 6).

## Discussion

The present study showed that prophylactic bilateral C4/5 foraminotomy did not contribute to the enlargement of the bony spinal canal parameters, including the GP and the LIA. However, prophylactic bilateral C4/5 foraminotomy effectively resulted in a larger postoperative CSA in the patients without postoperative C5 palsy. However, prophylactic C4/5 foraminotomy did not specifically prevent postoperative C5 palsy due to foraminal stenosis at C4/5.

Many studies have reported the effectiveness of prophylactic bilateral C4/5 foraminotomy in preventing C5 palsy after LP.^7-^^9^ Katsumi et al.^9^ reported that the incidence of C5 palsy in 141 patients who underwent open-door LP with prophylactic bilateral C4/5 foraminotomy was 1.4%, which was significantly lower than that in patients without foraminotomy (6.4%). In contrast, several studies have reported that prophylactic C4/5 foraminotomy did not reduce the incidence of C5 palsy.^10–12^ Liu et al.^10^ reported that the incidence of C5 palsy in 70 patients who underwent open-door LP with prophylactic bilateral C4/5 foraminotomy was 5.7%. Therefore, it remains controversial whether prophylactic C4/5 foraminotomy effectively prevents postoperative C5 palsy. In the present study, although the cut-off values of the bony spinal canal parameters in the F (+) group were similar to those in the F (–) group, foraminotomy resulted in a larger postoperative CSA at C4/5 and C5/6 in the patients without postoperative C5 palsy. The smaller postoperative CSA in the F (–) group compared with the F (+) group may be due to the inhibition of the expansion of the dural sac at C5, which has the shortest nerve root of the cervical nerves.^23,24^

Uematsu et al.^25^ reported that postoperative radiculopathy can be prevented by creating LIA after expansion of less than 60°and placing the bony gutter at the medial side of the intervertebral joint. Similarly, the mean cut-off value of the laminar inclination angle was 62° in the F (−) group in the present study. In contrast, Tsuji et al.^26^ reported that a cutoff value of 53.5° for the LIA predicted the development of C5 palsy in 190 patients who underwent open-door LP. The reason for the larger mean cut-off value of the LIA in the F (−) group in the present study than in the study by Tsuji et al.^26^ may be due to the difference in the LP procedures; double-door LP vs. open-door LP. Bilateral C4/5 foraminotomy did not contribute to the enlargement of the LIA in the present study, as the mean LIA at C4-C6 in the F (+) P (−) subgroup (57°-59°) were similar to those in the F (−) P (−) subgroup.

Bilateral C4/5 foraminotomy did not reduce the incidence of postoperative C5 palsy in the present study. A meta-analysis reported that the incidence of postoperative C5 palsy over the last decade was 6.3% (range, 1-29%).^27^ In the present study, the incidences of postoperative C5 palsy in the F (+) and F (−) groups were very high (18.8% and 14.7%, respectively), as we gradually extended the bony spinal canal over time in this case series to achieve sufficient expansion of the spinal cord and the dural sac in both the F (+) and F (−) groups, and to examine the effects of C4/5 foraminotomy in the F (+) group (Figure 3). These bony spinal canal parameters, including the GP and the LIA, were not mentioned in detail in the previous studies that investigated the effectiveness of C4/5 foraminotomy in preventing postoperative C5 palsy. There is currently no definitive method for preventing C5 palsy after LP, as the degree of bony spinal canal expansion cannot be precisely controlled. However, surgeons should make the LIA at C4-C6 in the F (+) group, or the LIA at C4 and C5 and the GP at C6 in the F (−) group, carefully to avoid C5 palsy from the results of the RR.

Preoperative foraminal stenosis at C4/5 has frequently been reported as a risk factor for C5 palsy after posterior cervical spine surgery.^19–21^ Lee et al.^21^ reviewed 116 patients who underwent open-door LP for CSM and reported that a foraminal diameter at C4/5 of less than 2 mm was significantly related to C5 palsy in the binary logistic regression test. In the present study, the patients with postoperative C5 palsy who had undergone C4/5 foraminotomy had a similar mean preoperative transverse foraminal diameter at C4/5 (2.8 mm) to that in the patients with postoperative C5 palsy who had not undergone C4/5 foraminotomy (2.3 mm). Therefore, C4/5 foraminotomy did not specifically prevent postoperative C5 palsy due to foraminal stenosis at C4/5.

The most important limitation of this study was the small number of cases. A larger sample size is needed to confirm the significance and relevance of our findings.

In conclusions, C4/5 foraminotomy should not be recommended for avoidance of C5 palsy. Although the bony spinal parameters were similar between the F (+) and F (−) groups, the CSA in the F (+) group was significantly than that in the F (−) group in the patients without C5 palsy.

**Highlights**

- The present study evaluated the cut-off values of spinal canal parameters (including the gutter positions, laminar inclination angles, and cross-sectional areas of the dural sac) as risk factors for C5 palsy after posterior cervical spine surgery in patients who underwent prophylactic C4/5 foraminotomy (F (+) group) and those who did not undergo foraminotomy (F (–) group).
- The cut-off values for each spinal canal parameter as a risk factor for postoperative C5 palsy were similar in the F (+) group and the F (–) group.
- In the patients without postoperative C5 palsy, the mean postoperative cross-sectional areas of the dural sac at C4/5 and C5/6 in patients who underwent foraminotomy were significantly larger than in those who did not undergo foraminotomy .
- Prophylactic C4/5 foraminotomy did not specifically prevent postoperative C5 palsy due to foraminal stenosis at C4/5.
- Prophylactic C4/5 foraminotomy should not be recommended for avoidance of C5 palsy, as the incidence of postoperative C5 palsy were similar between the F (+) group and the F (−) group.

## Figures and Tables

**Table 1.** Summary of F (+) Group and F (–) Group

	F (+) group		F (–) group	
Number of cases	69		34	
Operative methods	51 LP	18 PDF	17 LP	17 PDF
Disease (CSM/OPLL; *n*)	33/18	5/13	13/4	3/14

*n*: number of patients.

LP: laminoplasty; PDF: posterior decompression and fusion; CSM: cervical spondylotic myelopathy; OPLL: ossification of the longitudinal ligament.

**Table 2.** Distributions of Patients in Each Perioperative Factor

	*CSM*				*OPLL*		
	P (–)	P (+)	*P*		P (–)	P (+)	*P*
F (+)	30	8	0.2527	F (+)	26	5	0.7076
F (–)	15	1		F (–)	14	4	
***Younger***				***Elderly***			
	P (–)	P (+)	*P*		P (–)	P (+)	*P*
F (+)	29	8	0.4647	F (+)	27	5	0.6840
F (–)	18	2		F (–)	11	3	
***C3-7 LP***				***C2-T1 PDF***			
	P (–)	P (+)	*P*		P (–)	P (+)	*P*
F (+)	44	7	>0.999	F (+)	12	6	0.4430
F (–)	15	2		F (–)	14	3	

*n*: Number of patients.

Fisher’s exact test was used for the statistical analysis.

CSM: cervical spondylotic myelopathy; OPLL: ossification of the posterior longitudinal ligament; younger: patients whose age at the surgery less than 67 years old; elderly: patients whose age at the surgery more than 67 years old underwent; LP: cervical laminoplasty; PDF: posterior decompression and fusion; F (+): patients who underwent bilateral C4/5 foraminotomy; F (–): patients who did not undergo bilateral C4/5 foraminotomy; P (+): patients who occurred C5 palsy; P (–): patients who did not occurred C5 palsy.

**Table 3.** Cut-off Values and AUC for the Occurrence of Postoperative C5 Palsy after Posterior Cervical Spine Surgery

	Cut-off value	AUC (95% CI)	Sensitivity	Specificity
***F (+) group:***				
Gutter position at C4	0.82	0.576 (0.374, 0.777)	0.538	0.625
Gutter position at C5	0.84	0.624 (0.464, 0.784)	0.615	0.661
Gutter position at C6	0.84	0.718 (0.552, 0.884)	0.692	0.750
Inclination angle of lamina at C4 (degrees)	59.6	0.743 (0.610, 0.876)	0.769	0.661
Inclination angle of lamina at C5 (degrees)	58.9	0.740 (0.598, 0.881)	0.769	0.607
Inclination angle of lamina at C6 (degrees)	62.4	0.666 (0.476, 0.856)	0.615	0.768
Cross-sectional area of the dural sac at C4/5 (mm^2^)	200.0	0.670 (0.497, 0.842)	0.846	0.500
Cross-sectional area of the dural sac at C5/6 (mm^2^)	189.5	0.646 (0.476, 0.815)	0.923	0.446
***F (–) group:***				
Gutter position at C4	0.81	0.721 (0.475, 0.967)	0.800	0.759
Gutter position at C5	0.80	0.614 (0.354, 0.873)	0.800	0.517
Gutter position at C6	0.89	0.645 (0.289, 1.00)	0.600	0.897
Inclination angle of lamina at C4 (degrees)	61.7	0.931 (0.838, 1.000)	1.000	0.793
Inclination angle of lamina at C5 (degrees)	62.4	0.897 (0.781, 1.000)	1.000	0.759
Inclination angle of lamina at C6 (degrees)	62.5	0.772 (0.494, 1.000)	0.800	0.828
Cross-sectional area of the dural sac at C4/5 (mm^2^)	197.5	0.862 (0.736, 0.989)	1.000	0.793
Cross-sectional area of the dural sac at C5/6 (mm^2^)	199.5	0.834 (0.672, 0.997)	0.800	0.793

Bold font indicates moderate accuracy (0.70-0.90) or high accuracy (0.90-1.0).

F (+) group: patients who underwent posterior cervical spinal surgery with bilateral C4/5 foraminotomy; F (–) group: patients who underwent posterior cervical spinal surgery without bilateral C4/5 foraminotomy; AUC: area under the receiver operating characteristic curves; CI: confidence intervals.

**Table 4.** Risk Factors of the Bony Spinal Canal Parameters for the Occurrence of C5 Palsy after Posterior Cervical Spine Surgery in Descending Order Based on the Risk Ratios in Each Group

Risk factor	Risk ratio
**F (+) group:**	
Inclination angle of lamina at C4 > 59.6°	4.598
Gutter position at C6 > 0.84	4.500
Inclination angle of lamina at C5 > 58.9°	3.854
Inclination angle of lamina at C6 > 62.4°	3.657
Gutter position at C5 > 0.84	2.489
Gutter position at C4 > 0.82	1.708
**F (–) group:**	
Inclination angle of lamina at C4 > 61.7°	∞
Inclination angle of lamina at C5 > 62.4°	∞
Inclination angle of lamina at C6 > 62.5°	10.667
Gutter position at C4 > 0.81	8.364
Gutter position at C6 > 0.88	7.840
Gutter position at C5 > 0.80	3.556

F (+) group: patients who underwent posterior cervical spinal surgery with bilateral C4/5 foraminotomy; F (–) group: patients who underwent posterior cervical spinal surgery without bilateral C4/5 foraminotomy.

**Table 5.** Spinal Canal Parameters on CT and MRI in Patients Who Underwent Posterior Cervical Spine Surgery with and without Foraminotomy

Patients with C5 palsy (n = 18)	F (+) P (+) subgroup (n = 13)	F (−) P (+) subgroup (n = 5)	*P*
Gutter position at C4	0.82 ± 0.10	0.84 ± 0.10	0.7298
Gutter position at C5	0.85 ± 0.07	0.82 ± 0.09	0.5871
Gutter position at C6	0.86 ± 0.08	0.85 ± 0.13	0.8823
Inclination angle of lamina at C4 (degrees)	63.7 ± 5.5	68.0 ± 5.0	0.1833
Inclination angle of lamina at C5 (degrees)	62.7 ± 5.8	67.4 ± 6.0	0.1529
Inclination angle of lamina at C6 (degrees)	63.9 ± 8.4	65.7 ± 9.4	0.7301
Cross-sectional area of the dural sac at C4/5 (mm^2^)	233.2 ± 53.9	222.8 ± 22.5	0.3004
Cross-sectional area of the dural sac at C5/6 (mm^2^)	222.7 ± 49.8	225.8 ± 33.8	0.9607
**Patients without C5 palsy (n = 85)**	**F** **(+) P** **(−) subgroup (n=** **56)**	**F** **(−) P** **(−) subgroup (n = 29)**	***P***
Gutter position at C4	0.82 ± 0.07	0.80 ± 0.08	0.1785
Gutter position at C5	0.85 ± 0.07	0.82 ± 0.08	0.2278
Gutter position at C6	0.80 ± 0.06	0.79 ± 0.07	0.1681
Inclination angle of lamina at C4 (degrees)	58.9 ± 5.3	57.7 ± 4.8	0.2700
Inclination angle of lamina at C5 (degrees)	57.8 ± 4.7	59.1 ± 4.1	0.2142
Inclination angle of lamina at C6 (degrees)	59.4 ± 6.0	58.2 ± 5.9	0.3397
Cross-sectional area of the dural sac at C4/5 (mm^2^)	202.3 ± 50.8	177.3 ± 40.6	**0.0181**
Cross-sectional area of the dural sac at C5/6 (mm^2^)	200.9 ± 42.5	178.9 ± 35.5	**0.0277**

The Mann–Whitney *U*-test was used for statistical analysis. Bold font indicates a significant *P-value*.

F (+) P (+) group: patients who underwent posterior cervical spinal surgery with bilateral C4/5 foraminotomy who developed postoperative C5 palsy; F (-) P (+) group: patients who underwent posterior cervical spinal surgery without bilateral C4/5 foraminotomy who developed postoperative C5 palsy; F (+) P (–) group: patients who underwent posterior cervical spinal surgery with bilateral C4/5 foraminotomy who did not develop postoperative C5 palsy; F (–) P (–) group: patients who underwent posterior cervical spinal surgery without bilateral C4/5 foraminotomy who did not develop postoperative C5 palsy; CT: computed tomography; MRI: magnetic resonance imaging.

**Table 6.** Preoperative Transverse Diameter of the Foramen at C4/5 in Patients Who Underwent Posterior Cervical Spine Surgery with and without Foraminotomy

Upper - extremities with C5 palsy	Foraminotomy (+) (14 upper - extremities)	Foraminotomy (−) (5 upper - extremities)	*P*
Foraminal diameter at C4/5 (mm)	2.8 ± 0.7	2.3 ± 0.5	0.1908
**Upper - extremities without C5 palsy**	**Foraminotomy (+)** (124 upper - extremities)	**Foraminotomy (−)** (63 upper - extremities)	
Foraminal diameter at C4/5 (mm)	3.3 ± 1.0	3.3 ± 1.2	0.6882

Mann–Whitney *U*-test was used for the statistical analysis.

## References

[1] Shiozaki T Otsuka H Nakata Y, . Spinal cord shift on magnetic resonance imaging at 24 hours after cervical laminoplasty. Spine. 2009;34(3):274-279. 10.1097/BRS.0b013e318194e275 19179922

[2] Nakashima H Imagama S Yukawa Y, . Multivariate analysis of C-5 palsy after cervical posterior fusion with instrumentation. J Neurosurg Spine. 2012;17(2):103-110. 10.3171/2012.4.SPINE11255 22632173

[3] Bydon M Macki M Aygun N, . Development of postoperative C5 palsy is associated with wider posterior decompression: An analysis of 41 patients. Spine J. 2014;13(6):2861-2867. 10.1016/j.spinee.2014.03.040 24704500

[4] Baba S Ikuta K Ikeuchi H, . Risk factor analysis for C5 palsy after double-door laminoplasty for cervical spondylotic myelopathy. Asian Spine J. 2016;10(2):298-308. 10.4184/asj.2016.10.2.298 27114771 PMC4843067

[5] Lim CH Roh SW Rhim SC Jeon SR. Clinical analysis of C5 palsy after cervical decompression surgery: Relationship between recovery duration and clinical and radiological factors. Eur Spine J. 2017;26(4):1101-1110. 10.1007/s00586-016-4664-4 27342613

[6] Nori S Aoyama R Ninomiya K, . Cervical laminoplasty of limited width prevents postoperative C5 palsy: A multivariate analysis of 263 muscle-preserving posterior decompression cases. Eur Spine J. 2017;26(9):2393-2403. 10.1007/s00586-017-5202-8 28660373

[7] Sasai K Saito T Akagi S Kato I Ohnari H Iida H. Preventing C5 palsy after laminoplasty. Spine. 2003;28(17):1972-1977. 10.1097/01.BRS.0000083237.94535.46 12973145

[8] Komagata M Nishiyama M Endo K Ikegami H Tanaka S Imakiire A. Prophylaxis of C5 palsy after cervical expansive laminoplasty by bilateral partial foraminotomy. Spine J. 2004;4(6):650-655. 10.1016/j.spinee.2004.03.022 15541697

[9] Katsumi K Yamazaki A Watanabe K Ohashi M Shoji H. Can prophylactic bilateral C4/C5 foraminotomy prevent postoperative C5 palsy after open-door laminoplasty? A prospective study. Spine. 2012;37(9):748-754. 10.1097/BRS.0b013e3182326957 21912316

[10] Liu G Reyes MR Riew KD. Why does C5palsy occur after prophylactic bilateral. Glob Spine J. 2017;7(7):696-702. 10.1177/2192568217699191 PMC5624369 28989850

[11] Ando M Tamaki T Matsumoto T, . Can postoperative deltoid weakness after cervical laminoplasty be prevented by using intraoperative neurophysiological monitoring? J Clin Monit Comput. 2019;33(1):123-132. 10.1007/s10877-018-0141-4 29667095

[12] Fujiwara Y Manabe H Izumi B Tanaka H Kawai K Tanaka N. The efficacy of intraoperative neurophysiological monitoring using transcranial electrically stimulated muscle-evoked potentials (TcE-MsEPs) for predicting postoperative segmental upper extremity motor paresis after cervical laminoplasty. Clin Spine Surg. 2016;29(4):E188-E195. 10.1097/BSD.0000000000000311 26147699 PMC4841153

[13] Fujiyoshi T Yamazaki M Kawabe J, . A new concept for making decisions regarding the surgical approach for cervical ossification of the posterior longitudinal ligament: The K-line. Spine. 2008;33(26):E990-E993. 10.1097/BRS.0b013e318188b300 19092610

[14] Takeuchi K Yokoyama T Numasawa T, . K-line (–) in the neck-flexed position in patients with ossification of the posterior longitudinal ligament is a risk factor for poor clinical outcome after cervical laminoplasty. Spine. 2016;41(24):1891-1895. 10.1097/BRS.0000000000001660 27120063

[15] Takeuchi K Yokoyama T Aburakawa S, . Axial symptoms after cervical laminoplasty with C3 laminectomy compared with conventional C3-C7 laminoplasty. A modified laminoplasty preserving the semispinalis cervicis inserted into axis. Spine. 2005;30(22):2544-2549. 10.1097/01.brs.0000186332.66490.ba 16284593

[16] Takeuchi K Yokoyama T Numasawa T Itabashi T Yamasaki Y Kudo H. A novel posterior approach preserving three muscles inserted at C2 in multilevel cervical posterior decompression and fusion using C2 pedicle screws. Eur Spine J. 2018;27(6):1349-1357. 10.1007/s00586-017-5402-2 29177553

[17] Nakano K Harata S Suetsuna F Araki T Itoh J. Spinous process-splitting laminoplasty using hydroxyapatite spinous process spacer. Spine. 1992;17(3):S41-S43. 10.1097/00007632-199203001-00009 1314432

[18] Takeuchi K Yokoyama T Wada KI Kudo H. Relationship between enlargement of the cross-sectional area of the dural sac and neurological improvements after cervical laminoplasty: Differences between cervical spondylotic myelopathy and ossification of the posterior longitudinal ligament. Spine Surg Relat Res. 2019;3(1):27-36. 10.22603/ssrr.2018-0008 31435548 PMC6690118

[19] Katsumi K Yamazaki A Watanabe K Ohashi M Shoji H. Analysis of C5 palsy after cervical open-door laminoplasty: Relationship between C5 palsy and foraminal stenosis. J Spinal Disord Tech. 2013;26(4):177-182. 10.1097/BSD.0b013e31823db346 22124424

[20] Kurakawa T Miyamoto H Kaneyama S Sumi M Uno K. C5 nerve palsy after posterior reconstruction surgery: Predictive risk factors of the incidence and critical range of correction for kyphosis. Eur Spine J. 2016;25(7):2060-2067. 10.1007/s00586-016-4548-7 27055443

[21] Lee HJ Ahn JS Shin B Lee H. C4/5 foraminal stenosis predicts C5 palsy after expansive open-door laminoplasty. Eur Spine J. 2017;26(9):2340-2347. 10.1007/s00586-017-5077-8 28432435

[22] Vanagas G. Receiver operating characteristic curves and comparison of cardiac surgery risk stratification systems. Interact Cardiovasc Thorac Surg. 2004;3(2):319-322. 10.1016/j.icvts.2004.01.008 17670248

[23] Shinomiya K Okawa A Nakao K, . Morphology of C5 ventral nerve rootlets as part of dissociated motor loss of deltoid muscle. Spine. 1994;19(22):2501-2504. 10.1097/00007632-199411001-00002 7855672

[24] Alleyne CH Cawley CM Barrow DL Bonner GD. Microsurgical anatomy of the dorsal cervical nerve roots and the cervical dorsal root ganglion/ventral root complexes. Surg Neurol. 1998;50(3):213-218. 10.1016/S0090-3019(97)00315-7 9736081

[25] Uematsu Y Tokuhashi Y Matsuzaki H. Radiculopathy after laminoplasty of the cervical spine. Spine. 1998;23(19):2057-2062. 10.1097/00007632-199810010-00004 9794049

[26] Tsuji T Matsumoto M Nakamura M, . Factors associated with postoperative C5 palsy after expansive open-door laminoplasty: Retrospective cohort study using multivariable analysis. Eur Spine J. 2017;26(9):2410-2416. 10.1007/s00586-017-5223-3 28733721

[27] Wang T Wang H Liu S Ding WY. Incidence of C5 nerve root palsy after cervical surgery: A meta-analysis for last decade. Medicine. 2017;96(45): e8560. 10.1097/MD.0000000000008560 29137073 PMC5690766

